# The association between sharing something difficult on social media and mental well-being among adolescents. Results from the “LifeOnSoMe”-study

**DOI:** 10.3389/fpsyg.2022.1026973

**Published:** 2022-11-23

**Authors:** Bjarte Kysnes, Gunnhild Johnsen Hjetland, Ellen Haug, Ingrid Holsen, Jens Christoffer Skogen

**Affiliations:** ^1^Department of Health Promotion and Development, University of Bergen, Bergen, Norway; ^2^Department of Health Promotion, Norwegian Institute of Public Health, Bergen, Norway; ^3^Centre for Evaluation of Public Health Measures, Norwegian Institute of Public Health, Oslo, Norway; ^4^Alcohol and Drug Research Western Norway, Stavanger University Hospital, Stavanger, Norway; ^5^Department of Teacher Education, NLA University College, Bergen, Norway

**Keywords:** self-disclosure, adolescence, well-being, social media, social support

## Abstract

**Introduction:**

Social media use is part of everyday life for adolescents. Over the last decade, concerns about the potential negative effects of social media use on mental health and well-being has been raised. Possible positive effects of social media use have to a lesser extent been explored. However, some studies have found associations between social support on social media and well-being. Self-disclosing thoughts and feelings on social media might provide social support and positively affect well-being. The current study aimed to explore adolescents’ experiences with sharing something difficult on social media and the association with well-being.

**Methods:**

The survey data in this cross-sectional study were collected from a sample of 2023 adolescents from senior high schools (mean age 17.4, 55.6% females). Mental well-being was measured using the Warwick-Edinburgh Mental Well-being scale (WEMWBS). Multiple linear regression adjusting for age, gender, social media frequency/duration of use, and the number of close friends was used to explore the association of sharing something difficult on social media and mental well-being.

**Results:**

The findings indicated that sharing something difficult on social media, either with a few friends/family members or in a public post, was associated with lower well-being. Perceived social support (easier to talk about, received support, positive experience) after sharing something difficult was associated with higher well-being. Females reported sharing something difficult more than boys, but no interaction effect of gender was found in the associations between private or public sharing and well-being.

**Discussion:**

The results indicate that social media can serve as a supportive environment for adolescents. Future research may want to explore additional aspects of adolescents’ social media use, sharing, and well-being, such as differences in public and private sharing. Such contributions will increase the knowledge base of optimal ways to seek social support through social media.

## Introduction

Social media is defined as “mobile and web-based technologies that create highly interactive platforms via which individuals and communities share, co-create, discuss, and modify user-generated content” (Kietzmann et al., 2011). Social media appear to be fully integrated in adolescents’ life. On average, 77% of 15-16-year-olds in 19 EU countries reported using social media daily (Smahel et al., 2020). Since the introduction of social media, time spent on social media has increased rapidly, and 88% of adolescent girls and 70% of adolescent boys at Norwegian senior high schools reported spending more than 1 h on social media each day in 2021 (Bakken, 2021). Further, a recent cross-national study including 29 countries showed that an average of 34% of adolescents reported using social media “almost all the time throughout the day” (Boer et al., 2020). The increase in social media use among adolescents has caused concerns about potential negative impacts, as evident by the increasing number of research reviews on social media and adolescents’ mental health and well-being (Best et al., 2014; Keles et al., 2019; Boer et al., 2020; Odgers and Jensen, 2020; Orben, 2020; Schønning et al., 2020; Webster et al., 2021; Valkenburg et al., 2022). Valkenburg et al. (2022) point at evidence suggesting that social media use is weakly associated with higher levels of both mental illness and higher levels of well-being. This apparent contradiction led the authors to argue that these two outcome measures should be examined separately. Moreover, several researchers propose that social interactions and the quality of social media use may be more strongly associated with mental health and well-being than the quantity of social media use (Orben, 2020; Schønning et al., 2020; Liu et al., 2022; Valkenburg et al., 2022). Research investigating the potential positive effects of social media use on adolescent mental health has been limited (Schønning et al., 2020). Such a focus is relevant as adolescence is viewed as an important period for emotional and social development (Patton et al., 2016).

Perceived social support means that the individual perceives that support would be available if needed (Barrera, 1986). Perceived social support has been positively associated with well-being and health (Cohen and Wills, 1985; Barrera, 1986; Taylor, 2007; Chu et al., 2010; Thoits, 2011). A recent longitudinal study on adolescents showed positive associations between perceived social support, especially from friends, and well-being outcomes in early adulthood (Jakobsen et al., 2022). This protective effect can be understood from the perspective of the relational regulation theory, which was introduced to explain the beneficial effects of perceived social support on mental health (Lakey and Orehek, 2011). The theory defines relational regulation as a “desired affect, action, or thought that results from interaction with or thinking about specific other people” (Lakey and Orehek, 2011). Social media provides opportunities for such relational interactions. Several reviews and studies have indicated that social media provides feedback and interactions that could promote perceived social support (Best et al., 2014; Kross et al., 2021), and subsequently contribute as a positive factor to adolescents’ mental health and well-being (Best et al., 2014; Quinn, 2019; Webster et al., 2021).

An important aspect of social interaction is sharing feelings and difficulties. Self-disclosure has been defined as “the intentional communication of information about the self to another person or group of people” (Masur, 2019). It has been argued that some of the main benefits of disclosing personal upsetting events or problems are the reactions from those shared with, and that the disclosure may lead to social support (Derlega et al., 1993). Some researchers have also used “social support seeking” as a similar term, which implies to a greater degree a way of coping with life stressors and difficulties (Frison and Eggermont, 2015). Self-disclosure may have different purposes. Self-disclosure can serve different relationship goals, such as relational development or social validation (Derlega et al., 1993). Achieving relational development by seeking to increase relational intimacy and closeness to another individual might drive the disclosure (Luo and Hancock, 2020). Social validation reflects the feedback received by others, which may motivate self-disclosure (Derlega et al., 1993). Furthermore, in self-disclosure theory, it is common to distinguish between dyadic or small group interactions, and one-to-many communication (Masur, 2019). One-to-many communication may be highly relevant in social media, as social media has made it effortless to share information through public status updates or stories. However, some studies have suggested that sharing personal feelings, concerns and intimate information is more frequent in private messaging compared to public status updates (Bazarova and Choi, 2014; Masur and Scharkow, 2016).

Adolescents who spend more time on social media may also be more likely to share personal feelings and difficulties (self-disclose) with others on social media platforms. For example, in 2018, 68% of US adolescents reported that social media made them feel that people supported them through difficult times (Pew Research Center, 2018b), implying that they previously might have shared something difficult. Moreover, in a recent qualitative study, adolescents saw social media as having a positive influence on their mental health because it allowed them to seek social support from friends, and some noted that it was easier to talk about difficulties through social media (Hjetland et al., 2021).

The impact of self-disclosure and social support seeking on social media on mental health and well-being is not established. Social support seeking through social media has been associated both with higher levels of depressed mood (Frison and Eggermont, 2015) and with higher levels of online social well-being (Huang, 2016), while some have argued that self-disclosure has no direct effect on well-being (Lee et al., 2013; Zhang, 2017). Nevertheless, the same studies reported that both seeking social support (Frison and Eggermont, 2015) and self-disclosure (Lee et al., 2013; Huang, 2016; Zhang, 2017) on social media were positively associated with perceived social support. Furthermore, perceived social support through Facebook has been shown to be associated with a decrease in depressed mood (Frison and Eggermont, 2015), as well as an increase in well-being (Lee et al., 2013; Zhang, 2017). Thus, perceived social support might be imperative for a positive impact of self-disclosure. This is in line with a theoretical framework by Luo and Hancock (2020), suggesting that self-disclosure on social media can affect psychological well-being through various mechanisms, such as perceived social support.

Most previous studies on self-disclosure and social support on social media have focused on university students, and much of the research has been limited to exploring Facebook as the social media platform (Lee et al., 2013; Frison and Eggermont, 2015; Frison and Eggermont, 2016; Huang, 2016; Zhang, 2017; Gilmour et al., 2020). YouTube, Instagram, Snapchat, and TikTok have been reported to be more popular than Facebook among adolescents (Pew Research Center, 2018a; Medietilsynet, 2020). Hence, rather than examining Facebook use only, social media platforms, in general, is of strong interest. Early research on self-disclosure suggested that males tend to disclose personal information or concerns less than females (Jourard, 1971; Derlega et al., 1981). Similar results have been found in more recent research, for offline and online self-disclosure among adolescents (Valkenburg et al., 2011). Gender differences in social support has also been found among adolescents, suggesting that females perceive higher levels of support than males (Bokhorst et al., 2010). Further, a meta-analysis showed that females receive more social support than males on social media (Tifferet, 2020). Females’ increased activity on social media compared to males has been suggested to explain higher level of perceived social support (Tifferet, 2020). Adolescent girls have reported more frequent use of social media than boys (Bakken, 2021). However, a European report showed that gender differences among adolescents are small for the use of social media sites (Smahel et al., 2020). The abovementioned meta-analysis (Tifferet, 2020) did not differentiate between age groups, and research on adolescents and gender differences in social support on social media seems limited. Furthermore, studies investigating gender differences in social media use and associations with well-being have been lacking in the research literature (Schønning et al., 2020).

Against this backdrop, the present study aimed to explore whether:

- sharing something difficult on social media is associated with well-being among adolescents
- perceived social support after sharing something difficult on social media is associated with well-being among adolescents
- there are gender differences in the associations between sharing something difficult on social media and well-being

## Materials and methods

This cross-sectional study was based on data from the first wave of a survey conducted in the autumn of 2020, “LifeOnSoMe”-Study (Skogen et al., 2022). The survey was a collaboration between the Norwegian Institute of Public Health, Bergen municipality, and Vestland County Council. The study used a web-based questionnaire, and the participants completed the questionnaires during school hours with teachers present in class.

### Participants

Invitations to participate were sent to all 14 senior high schools in Bergen Municipality. In the 12 schools that accepted the invitation, all pupils aged 16 or more were invited (*n =* 3,959), of which 2,116 (53.4%) pupils participated. Those who did not reply to the gender and age questions in the survey were excluded from the analysis (*n* = 71). Those who indicated non-binary gender were excluded due to very low numbers (*n* = 13) and privacy concerns. Nine responses were excluded as they were duplicates, resulting in a final sample of 2023 (51.1%), of which 899 (44.4%) were males and 1,124 (55.6%) were females. The age range was 16–21, and the average age was 17.3 years (*SD =* 0.9) for males and 17.4 years (*SD =* 0.9) for females (Table 1).

**Table 1:** Descriptive statistics comparing gender for both control and main variables.

Variables (valid observations)	^1^Males, N = 899	^1^Females, N = 1,124	^1^Total, N = 2023	^2^p-value
Age (N = 2023; 100%)	17.3 (0.9)	17.4 (0.9)	17.4 (0.85)	=0.130
Number of close friends (N = 2017; 99.7%)				=0.290
None	25 (2.8%)	20 (1.8%)	45 (2.2%)	
One	56 (6.3%)	67 (6.0%)	123 (6.1%)	
Two or more	813 (90.9%)	1,036 (92.3%)	1849 (91.7%)	
Frequency use of social media (N = 2007; 99.2%)				<0.001
Rarer than every day	37 (4.2%)	16 (1.4%)	53 (2.6%)	
Every day	189 (21.3%)	174 (15.5%)	363 (18.1%)	
Several times a day	439 (49.5%)	582 (51.9%)	1,021 (50.9%)	
Almost all the time	221 (24.9%)	349 (31.1%)	570 (28.4%)	
Duration of social media use (N = 1998; 98.8%)				<0.001
Less than 30 min	35 (4.0%)	11 (1.0%)	46 (2.3%)	
30 min – 1 h	101 (11.5%)	65 (5.8%)	166 (8.3%)	
1–2 h	184 (20.9%)	170 (15.2%)	354 (17.7%)	
2–4 h*	326 (37.0%)	402 (36.0%)	728 (36.4%)	
4–5 h	134 (15.2%)	284 (25.4%)	418 (20.9%)	
More than 5 h	101 (11.5%)	185 (16.6%)	286 (14.3%)	
Public sharing (N = 1950; 96.4%)				=0.019
Yes	52 (6.1%)	99 (9.0%)	151 (7.7%)	
No	803 (93.9%)	996 (91.0%)	1799 (92.3%)	
Private sharing (N = 1944; 96.1%)				<0.001
Yes	202 (23.6%)	409 (37.6%)	611 (31.4%)	
No	653 (76.4%)	680 (62.4%)	1,333 (68.6%)	
Total score WEMWBS (N = 2018; 99.8%)	51.8 (10.0)	46,2 (9.4)	48.7 (10.0)	<0.001

1 Mean (SD); n (%).

2 Independent t-test; Pearson’s Chi-squared test.

* Values 2–3 h and 3–4 h has been collapsed due to errors in answer options in the electronic survey.

### Instruments

#### Measure of mental well-being

The Warwick-Edinburgh Mental Well-being Scale (WEMWBS) was used to assess the level of mental well-being. This instrument aims at measuring well-being, conceptualized broadly to include affective-emotional aspects, psychological functioning, and cognitive-evaluative dimensions (Tennant et al., 2007). The WEMWBS consists of 14 items addressing positive aspects of mental health, and the participants were asked to indicate how much each statement pertained to them based on the previous 2 weeks. They responded to the statements using a 5-point Likert scale (1 = none of the time, 2 = rarely, 3 = some of the time, 4 = often, 5 = all of the time; Tennant et al., 2007). Some examples of the statements are: “I’ve been feeling optimistic about the future,” “I’ve been dealing with problems well,” and “I’ve been feeling loved.” The sum score is calculated by summarizing the scores on each item, with the total score ranging from 14 to 70. The higher the score, the higher level of mental well-being (Tennant et al., 2007). A validated Norwegian version of WEMWBS was used in the present study (Smith et al., 2017). Cronbach’s α was 0.93 in the current study, indicating a high internal consistency.

#### Sharing and perceived social support

Questions related to sharing something difficult on social media and perceived social support were based on a qualitative study using focus group interviews among adolescents in senior high schools (Hjetland et al., 2021). Examining participants’ experiences with sharing something difficult on social media included two introductory questions: “Have you ever shared something difficult through a story, a post, or similar, which was public or visible to others than your closest friends?” (public sharing) and “Have you ever shared something difficult with one or a few friends/family members through social media?” (private sharing). Response alternatives were “yes” or “no,” coded as 1 and 0, respectively. Those responding “yes” on one or both questions were presented with three statements: (1) “It was easier to talk about the difficulties in real life afterwards” (2) “I received support from friends and people I know afterwards” (public sharing)/“I received support from those I shared it with afterwards” (private sharing), and (3) “It was a positive experience to share the difficult issue on social media.” The participants responded to the statements on a Likert scale ranging from 1 = “not at all” to 5 = “to a great extent.” Responses were recoded to three alternatives, 1–2 = “not at all/to a little extent,” 3 = “to some extent,” and 4–5 = “A lot/very much” for the purposes of the present study.

#### Control variables

Due to their potential influence on well-being and social support, frequency/duration of social media use (Boer et al., 2020; Schønning et al., 2020), gender (Zhang, 2017; Liu et al., 2018; Tifferet, 2020), age (Liu et al., 2018), and the number of friends (Helliwell and Huang, 2013) were included as control variables. To assess frequency of social media use, the respondents were asked how often they use social media. The response alternatives were “Almost never,” “Several times a month, but rarer than every week,” “1–2 times a week,” “3–4 times a week,” “5–6 times a week,” “Every day,” “Several times a day” and “Almost all the time.” Responses were recoded to 1 = “Less than every day,” 2 = “Every day,” 3 = “Several times a day” and 4 = “Almost all the time.” In addition, the respondents were asked about the duration of social media use: “On the days that you use social media, approximately how much time do you spend using them?.” Response alternatives were “<30 min,” “between 30 min and 1 h,” 1–2 h,” “2–4 h,” “4–5 h” and “>5 h,” making a scale range from 1 to 6.

Age categories were 16, 17, 18, 19, 20, and 21 years. Response alternatives on gender question were “girl,” “boy” and “non-binary.” Finally, the respondents were asked about number of close friends, with the alternatives 1 = “none,” 2 = “one,” and 3 = “two or more.”

### Ethical considerations

The data collection was conducted according to the guidelines of the Declaration of Helsinki and was approved by the Regional Ethics Committee (REK) in Norway (REK #65611). The adolescents invited were 16 years or older and were able to consent to participate on their own behalf.

### Data analyses

Descriptive statistics for the main variables are presented stratified for gender in Table 1. Males and females were compared in terms of WEMWBS and age using independent t-tests. The comparison of males and females in public and private sharing, number of close friends, frequency of social media use, and duration of social media use was done using the Chi-square test for independence. The overall response rates for each statement related to social support (‘easier to talk about’, ‘received support’ and ‘positive experience’) after answering about private and public sharing are presented in Table 2. Assumptions were checked for each analysis, including minimum expected cell frequency for Chi-square tests, normal distribution, and homogeneity of variance in t-tests (Levene’s test for equality of variance). Additionally, we estimated the polychoric correlations between the three statements on social support asked after affirming public and/or private sharing.

**Table 2:** Frequency distribution of responses on the three statements after answering “Yes” on the two questions about sharing something difficult.

Variables (valid observations)	Not at all n (%)	Some extent n (%)	Great extent n (%)
Public sharing (N = 151)			
It was easier to talk about the difficulties in real life afterwards (N = 146; 96.7%)	38 (26.0%)	60 (41.1%)	48 (32.9%)
I received support from friends and people I know afterwards (N = 145; 96.0%)	12 (8.3%)	37 (25.5%)	96 (66.2%)
It was a positive experience to share the difficult issue on social media (N = 147; 97.4%)	32 (21.8%)	56 (38.1%)	59 (40.1%)
Private sharing (N = 611)			
It was easier to talk about the difficulties in real life afterwards (N = 600; 98.2%)	80 (13.3%)	210 (35.0%)	310 (51.7%)
I received support from those I shared it with afterwards (N = 602; 98.5%)	24 (4.0%)	107 (17.8%)	471 (78.2%)
It was a positive experience to share the difficult issue on social media (N = 596; 97.5%)	70 (11.7%)	195 (32.7%)	331 (55.5%)

**Figure 1:**
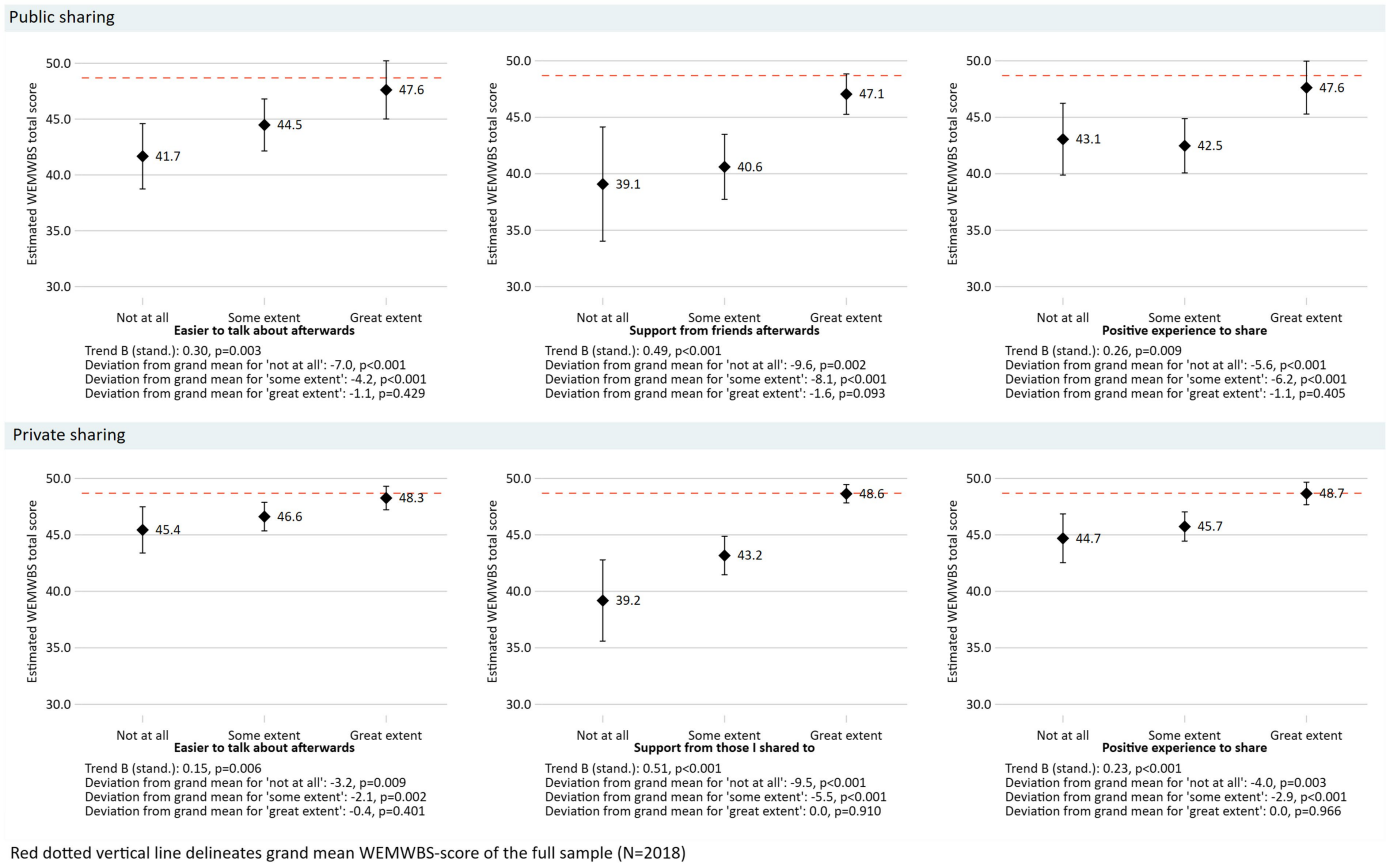
Results from bivariate linear regression analyses for public and private sharing and the related social support statements. WEMWBS is the dependent variable. Estimated total scores across response options. B (stand.) was computed using a Z-scored (mean 0; standard deviation 1) dependent variable.

Bivariate linear regression with WEMWBS as the dependent variable was used to investigate relationships with the variables public sharing and private sharing, and their three related statements. In the multiple linear regression analysis, the relationship between WEMWBS and sharing were adjusted for gender and the other control variables (Table 3). The dependent variable WEMWBS was also Z-scored to ease interpretation of the association strength. Interaction analysis was used to examine a potential gender moderation in the associations between WEMWBS and sharing using likelihood ratio tests to compare models with and without gender×sharing. The results from individual bivariate linear regression for association between the three statements on social support after each sharing option and WEMWBS are presented in Table 4 (unstandardized and standardized trend coefficients) and Figure 1 (estimated unstandardized WEMWBS total scores across response options). Assumptions were checked for each analysis, including normality, linearity, homoscedasticity, and examining residuals. No major deviations or violations were found. The IBM SPSS Windows version 26 and Stata version 17.0 software (Statacorp, 2021) was used for data analysis.

**Table 3:** Results from multiple regression analyses adjusting for covariates.

Variables		Public sharing				Private sharing		
	B (stand.)	B	95% CI	*p*-value	B (stand.)	B	95% CI	*p*-value
Unadjusted	−0.44	−4.38	−6.02, −2.73	<0.001	−0.20	−2.00	−2.95, −1.05	<0.001
Adjusted for:								
Age	−0.43	−4.35	−6.00, −2.71	<0.001	−0.20	−2.01	−2.96, −1.06	<0.001
Gender	−0.38	−3.80	−5.38, −2.23	<0.001	−0.11	−1.12	−2.04, −0.19	=0.018
Close friends	−0.40	−4.41	−6.02, −2.80	<0.001	−0.22	−2.25	−3.18, −1.32	<0.001
Frequency, SoMe use	−0.43	−4.31	−5.96, −2.67	<0.001	−0.19	−1.95	−2.91, −0.99	<0.001
Duration SoMe, use	−0.38	−3.84	−5.47, −2.20	<0.001	−0.17	−1.68	−2.63, −0.73	=0.001
Fully adjusted	−0.36	−3.57	−5.11, −2.02	<0.001	−0.13	−1.31	−2.22, −0.41	=0.005

B (stand.) was computed using a Z-scored (mean 0; standard deviation 1) dependent variable. The association between WEMWBS and sharing were adjusted for each covariate separately. WEMWBS is the dependent variable.

**Table 4:** Results from bivariate linear regression analyses for public and private sharing and the related social support statements.

Variables	B (stand.)	B	CI 95%	*p*-value
Public sharing				
It was easier to talk about the difficulties in real life afterwards	0.30	2.98	1.04, 4.92	=0.003
I received support from friends and people I know afterwards	0.49	4.87	2.59, 7.15	<0.001
It was a positive experience to share the difficult issue on social media	0.26	2.63	0.67, 4.58	=0.009
Private sharing				
It was easier to talk about the difficulties in real life afterwards	0.15	1.47	0.42, 2.53	=0.006
I received support from those I shared it with afterwards	0.51	5.09	3.71, 6.47	<0.001
It was a positive experience to share the difficult issue on social media	0.23	2.27	1.20, 3.33	<0.001

B (stand.) was computed using a Z-scored (mean 0; standard deviation 1) dependent variable. WEMWBS is the dependent variable.

#### *Post-hoc* analyses

In *post-hoc* analyses, a potential gender moderation between the three statements on social support and wellbeing was tested for the participants endorsing private and/or public sharing. Likelihood ratio tests were used to compare models with and without the interaction term gender×statement.

### Missing values

Initial analyses included checking the distribution of errors or missing values in the data set. The WEMWBS had some missing values (from 0.4 to 2.8% for the individual items). Five participants had 100% missing values on the WEMWBS, while 111 had one or more missing items (of which 79% were missing one item). The total score for the WEMWBS was calculated for those with <100% missing items by summarizing their scores on all answered items, divided by the number of answered items, and multiplied by 14. The study had a high number of participants (*N* = 2023), which means that the results probably would not be affected to a great degree by the relatively few missing values (Tabachnick and Fidell, 2013). To maximise the number of respondents in each regression model, pairwise deletion was employed, to account for missingness on other variables. Across all variables, the fractions of valid observations were more than 95%.

## Results

In total, 89% of the respondents used social media for more than 1 h per day (Table 1). Significant gender differences were found in frequency of use, with 93% of the females reporting using social media for more than 1 h per day, compared to 85% of males. Males reported a significantly higher level of well-being than females (*p* < 0.001). For public sharing, 9.0% of females and 6.1% of males reported this activity (*p* = 0.019), while 38% of females had shared something difficult in private, compared to 24% of the males (*p* < 0.001; Table 1).

The response rates for each statement related to private and public sharing are presented in Table 2. For both public and private sharing, the majority of the respondents indicated that they to a “great extent” received support after sharing (66 and 78%, respectively). For public sharing, 26% did not experience it easier to talk about the difficulties in real life afterwards, compared to 13% for private sharing. For the three related statements after affirming public sharing, the polychoric correlation ranged between 0.55 (for ‘easier to talk about’ and ‘received support from friends’) and 0.64 (for ‘easier to talk about’ and ‘positive experience to share’). Likewise, the polychoric correlation of the three statements after affirming private sharing ranged between 0.58 (for ‘easier to talk about’ and ‘received support from those I shared it with’) and 0.67 (for ‘easier to talk about’ and ‘positive experience to share’). All correlations were statistically significant at *p* < 0.001.

Table 3 shows the results from the multiple regressions analyses. The results showed that those who had shared something difficult in public reported lower well-being than those who had never shared something difficult in public [B (stand.) = −0.44, *p* < 0.001]. Private sharing was also associated with lower well-being [B (stand.) = −0.20, *p* < 0.001]. In the fully adjusted analyses, the effect size was small to medium for public sharing [B (stand.) = −0.36] and small for private sharing [B (stand.) = −0.13]. Compared to the unadjusted estimates, the changes in point estimates were small. The most pronounced differences were seen when adjusting for gender. We also investigated the potential interaction between gender and public/private sharing. The interaction analyses indicated no interaction effect for gender on the associations between public sharing (*p* = 0.839 for interaction term) and private sharing (*p* = 0.295), and WEMWBS. The *post-hoc* likelihood ratio tests for potential gender moderation between statements related to social support and well-being were also non-significant (*p*-values ranging between 0.287 and 0.861), and no support for differences in the reported associations were identified.

Results from bivariate linear regression analyses for public and private sharing and the related social support statements are presented in Table 4. For those who reported public sharing, higher levels of receiving support after sharing was associated with increased well-being [B (stand.) = 0.49, *p* < 0.001]. This was the highest point estimate of the three statements related to public sharing. For those who had shared something difficult in private, higher scores on receiving support were associated with higher well-being [B (stand) = 0.51, *p* < 0.001]. All variables showed significant associations (*p* < 0.05). For all of the six statements there was a positive monotonic relationship across response levels (‘not at all’, ‘some extent’ and ‘great extent’) and well-being (Figure 1). Also, for those endorsing response option ‘great extent’, there was no deviation from the grand mean of well-being in the study sample (Figure 1).

## Discussion

The current study aimed to explore adolescents’ experiences with sharing something difficult on social media and its relation to well-being, as previous research on this topic, to our knowledge, is modest. The results showed that having shared something difficult on social media, either privately (with one or a few friends or family members) or in a public post (public or visible to others than one’s closest friends), was associated with lower well-being among adolescents. Moreover, adjusting for covariates did not change the observed association between sharing and well-being substantially. Among those who had shared something difficult, higher scores on each of the three statements of social support (finding it easier to talk about the difficult in real life, having received social support, and viewing sharing as a positive experience) were associated with higher levels of well-being. In fact, those reporting receiving social support in any kind to a ‘great extent’ did not differ in the reported well-being compared to the overall sample. There were significant gender differences, with girls sharing more than boys, but no gender interaction was found on the associations between public or private sharing or the three following social support statements, and well-being.

The finding that sharing something difficult was associated with lower levels of well-being, may reflect that those who shared something difficult through social media also experience more difficulties and hence had lower well-being. Similarly, Frison and Eggermont (2015) found that social support seeking on Facebook predicted higher levels of depressed mood. However, if the social support seeking was followed by perceived social support, social support seeking was associated with lower levels of depressed mood (Frison and Eggermont, 2015). The present results show something similar: Among those who had shared something difficult on social media, higher scores on the social support statements were associated with higher well-being. The highest point estimate was found for the second statement, having received social support, with medium effect sizes for both public and private sharing. Nevertheless, all statements were associated with a higher level of well-being across public and private sharing. Thus, the present findings support the theoretical framework on self-disclosure on social media and well-being presented by Luo and Hancock (2020), stating that perceived social support through self-disclosure positively affects psychological well-being. The results also align with previous studies and reviews, showing that perceived social support on social media is associated with higher well-being (Best et al., 2014; Huang, 2016; Kross et al., 2021; Webster et al., 2021). Adolescence is viewed as an important period for acquiring emotional, social, economic, cognitive, and physical resources (Patton et al., 2016). Those resources could protect individuals against adverse health outcomes and promote good health later on (Morgan and Ziglio, 2010). In that regard, the current study suggests that receiving social support through social media could serve as an asset with the potential ability to protect adolescents against potential negative health outcomes and to promote well-being and good health.

About four times as many adolescents in the present study reported having shared something difficult with one or a few friends/family members, compared to public sharing. Similar results were found in a study among adults, where participants shared information more often in private messages than status updates (Masur and Scharkow, 2016). Moreover, various research has reported that personal feelings and intimate information are rarely disclosed in status updates or wall posts (Bazarova and Choi, 2014; Masur and Scharkow, 2016). This may indicate differences in the responses people receive after public and private sharing. Some suggest that disclosing negative thoughts and feelings in public status updates may receive fewer responses than via private messages (Ziegele and Reinecke, 2017). This might have been reflected in the present study. Among those who had shared privately, more than three out of four reported having received social support from those they shared it with, compared to two out of three for public sharing. Similarly, half of the respondents reported that it was easier to talk about the difficult issue in real life after sharing in private, compared to one third for public sharing. However, this comparison might be biased, considering that private sharing assumably are more likely to occur with the same people they talk with in real-life afterwards, compared to public sharing.

The findings in the current study suggest that significantly more females than males had shared difficulties on social media, both in public and private. This is in line with previous research on self-disclosure and gender differences, showing that males tend to disclose personal information or concerns to a lesser degree than females (Jourard, 1971; Derlega et al., 1981; Valkenburg et al., 2011). Gender did not, however, substantially alter the association between sharing and wellbeing or between social support and well-being, suggesting that these relationships were similar for both genders.

The current study investigated sharing on social media and did not differentiate between platforms like Instagram, Facebook, TikTok, Snapchat, etc. Previous studies have mainly focused on Facebook (Lee et al., 2013; Frison and Eggermont, 2015; Frison and Eggermont, 2016; Huang, 2016; Zhang, 2017; Gilmour et al., 2020), which has led to concerns about generalizability (Stoycheff et al., 2017). These concerns could be valid, as it has been noted that certain types of social support seeking might be more frequent in some social media platforms than others (Hayes et al., 2016). With that in mind, future research should further explore how different social media platforms are being used to share something difficult. This might broaden the knowledge base for researchers and health promotion practitioners to consider optimal ways of seeking social support through social media.

### Implications

The current findings show that social media can serve as an arena for receiving social support among adolescents and that perceived social support on social media is associated with higher levels of well-being. Sharing difficulties and receiving social support thus represent aspects of social media use that could positively impact on adolescents’ well-being. Considering the extensive use of social media among adolescents, social media might be an essential social arena where they can share difficulties that they otherwise would not have revealed or found challenging to share face-to-face. Sharing through social media may elicit immediate responses and social support digitally, and subsequent support in a face-to-face setting. However, social support seeking or self-disclosure without actually receiving social support has been associated with more depressive symptoms (Frison and Eggermont, 2015), as well as lower well-being in the present study. Future research should try to identify the best possible ways of sharing to elicit social support, thereby optimizing the positive aspects of sharing and reducing the negative ones. For instance, future research could examine how often adolescents share something difficult on social media, their motivations for sharing, and how this relates to social support and well-being, using both qualitative and quantitative approaches. Furthermore, it is likely that perceived social support will depend on the target of one’s sharing and/or who provides social support. The present study did not examine differences in perceived support from parents, friends, teachers, and classmates. It has been suggested that adolescents might perceive friend support as more helpful than parent support (Bokhorst et al., 2010), and perceived support from friends in adolescence has also been reported as most important for positive mental health in early adulthood (Jakobsen et al., 2022). In that respect, research that could illuminate differences in social support sources on social media among adolescents is warranted.

### Strengths and limitations

A strength of the current study was the investigation of specific aspects of social media use rather than the overall quantity of social media use, and its relation to well-being, which scholars have requested (Orben, 2020; Schønning et al., 2020; Valkenburg et al., 2022). Furthermore, another strength of this study was the use of a validated scale on well-being focusing on positive mental health, which has been missing in the literature on self-disclosure and social support among adolescents, and the literature on social media use. Finally, researchers have underscored the importance of investigating how social interactions on social media relates to other modes of interactions (Hamilton et al., 2022). The present study does just that by linking sharing of something difficult on social media to talking about it in real life afterwards.

The present study has some limitations. First, the cross-sectional approach prevents us from drawing conclusions about causality. The association between sharing something difficult and well-being may reflect that those who shared something difficult had initial struggles and hence reported lower well-being than those who had not shared difficulties. However, it cannot be ruled out that sharing something difficult on social media leads to lower well-being. The same applies to the three social support measures where the direction of the associations and causality is uncertain. Longitudinal studies on the topic are needed to gauge the causal relationship between sharing, social support, and mental well-being. Second, respondents were to subjectively interpret what sharing something difficult would imply, and there may have been a wide range in the severity or graveness of the difficulties shared by the participants. In a previous study, researchers measured specific disclosure types on social media, such as personal feelings, fears and concerns, relationship details and more, as well as disclosure frequency (Masur and Scharkow, 2016). This may serve as more specific measures. However, a similar measure as in the present study was used by Frison and Eggermont (2015) in their study on social support seeking on Facebook. Future research could look at differences in which difficulties are shared publicly and privately, and whether the type of the difficulty affects the nature of the response.

Third, the frequency of sharing was not investigated. One study showed that frequently talking about oneself on Facebook was negatively associated with perceived social support among young adults (Zhang, 2017). Although not fully transferable, a similar reference was made by Cozby (1973) for offline settings. He hypothesized that being either high or low in disclosure to significant others or to others in their social environment may be associated with negative mental health, compared to those described as high disclosers to significant others and medium disclosers to others in their environment.

Fourth, we used non-validated questions related to perceived social support. The formulation of the second statement on public sharing, “I received support from friends and people I know afterwards,” might have neglected potential support received by a greater online community and unknown people. This may have limited the results. Future research could investigate this more specifically. Nevertheless, the face validity of the social support measures was strengthened by initial focus group discussions and feedback from a resource group of adolescents (Hjetland et al., 2021).

Finally, the survey was conducted during the COVID-19 pandemic. This meant that adolescents were restricted from physical meetings, and presumably used mobile and data technology to a greater extent than before the pandemic. The amount of sharing something difficult through social media may have been different now or before the pandemic. However, the adolescents were responding to questions based on previous events. Even though the survey was conducted during the pandemic, the respondents might have had experiences with sharing something difficult prior to the pandemic which came to mind when answering the questions.

## Conclusion

The findings in the current study extend prior research on associations between perceived social support and well-being among adolescents by looking at the action of sharing something difficult on social media. The results showed that sharing something difficult on social media was associated with lower well-being. However, among those who had shared something difficult, perceived support after sharing was associated with higher well-being. Further, girls reported to share something difficult significantly more than boys. The findings indicate that social media may provide a supportive environment for adolescents, and that receiving support through social media could have a potential positive effect on adolescents’ well-being. Future research may want to seek greater knowledge on several aspect of social media use, sharing, social support, and well-being, such as different experiences of private and public sharing and how received social support depends on the target of one’s disclosure or the frequency of disclosure. This may contribute to find possible and/or optimal ways for seeking social support on social media.

## Data availability statement

The datasets presented in this article are not readily available because the data analyzed in this study is subject to the following licenses/restrictions. The Norwegian Health research legislation and the Norwegian Ethics Committee require explicit consent from the participants to transfer health research data outside of Norway. For the data employed in the present study, ethics approval was also contingent on storing the research data on secure storage facilities located at the Norwegian Institute of Public Health, which prevents us from providing the data as supplementary material or to transfer it to data repositories. Requests to access these datasets should be directed to JS, jens.christoffer.skogen@fhi.no.

## Ethics statement

The studies involving human participants were reviewed and approved by the Regional Committee for Medical Research Ethics Western Norway. Written informed consent from the participants’ legal guardian/next of kin was not required to participate in this study in accordance with the national legislation and the institutional requirements.

## Author contributions

JS: original conceptualization. BK and JS: formal analysis. BK: writing—original draft and revised the manuscript after feedback from GH, EH, IH, and JS. JS and GH: feedback on analytical approach and writing—review and editing. JS, GH, EH, and IH: writing, review and editing. All authors have made substantial contributions to the conception, the design of the work, and interpretation of results. All authors contributed to the article and approved the submitted version.

## Funding

This article was partly based on the work carried out by BK for his Master Thesis (Faculty of Psychology, University of Bergen; Bergen Open Research Archive) under supervision by GH, EH, IH, and JS. The present study is related to an innovative project on social media use and mental health and well-being among adolescents, led by Bergen municipality in Western Norway.

## Conflict of interest

The authors declare that the research was conducted in the absence of any commercial or financial relationships that could be construed as a potential conflict of interest.

## Publisher’s note

All claims expressed in this article are solely those of the authors and do not necessarily represent those of their affiliated organizations, or those of the publisher, the editors and the reviewers. Any product that may be evaluated in this article, or claim that may be made by its manufacturer, is not guaranteed or endorsed by the publisher.
